# 1191. The Implementation of Nasal MRSA PCR for Pneumonia and Association with Antibiotic Use and Clinical Outcomes

**DOI:** 10.1093/ofid/ofad500.1031

**Published:** 2023-11-27

**Authors:** Luke A Fenlon, Karen Fong, Emily S Spivak, Hannah Imlay

**Affiliations:** University of Utah, North Salt Lake City, Utah; University of Utah Health, Salt Lake City, Utah; University of Utah School of Medicine, Salt Lake City, UT; University of Utah Health, Salt Lake City, Utah

## Abstract

**Background:**

Nasal MRSA PCR testing has been successfully implemented at many centers and limits vancomycin exposure among hospitalized patients with pneumonia. There are few studies of a Best Practice Alert (BPA) to direct nasal MRSA PCR use and vancomycin de-escalation in the absence of other stewardship oversight. On 1/2/2020, nasal MRSA PCR and a BPA were implemented at our institution. Providers ordering vancomycin for patients with an indication of pneumonia or undifferentiated sepsis were directed to order a nasal MRSA PCR and recommended to stop vancomycin if negative (Figure 1). Our aim was to examine the impact of BPAs implementation on vancomycin duration among patients with suspected pneumonia.

**Figure:**
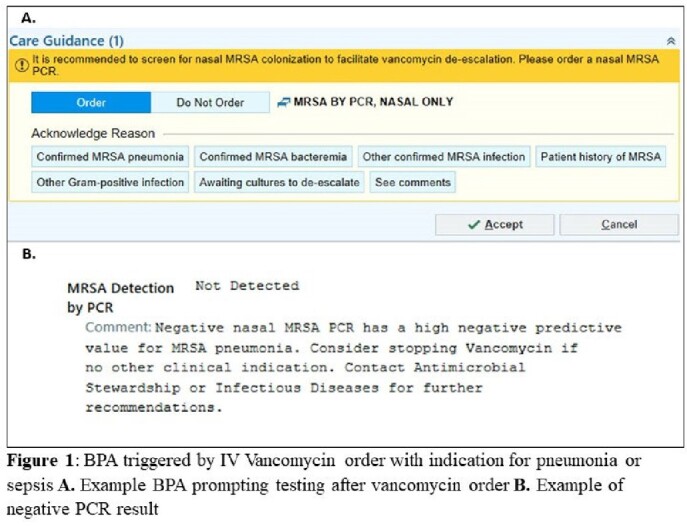

**Methods:**

A retrospective chart review was conducted among hospitalized patients without prior MRSA PCR performed during their admission who had a listed indication of pneumonia or sepsis at the time of vancomycin order. Patients were categorized into “pre” (9/17/2018 – 1/1/2020) and “post” (1/2/2020 – 3/20/2021) cohorts based on date of vancomycin order. Demographics, comorbidities, and antibiotic duration were electronically extracted. Uni- and multivariable logistic models were used to examine the proportion of patients in the pre and post cohorts whose vancomycin was stopped within 24 hours of initial order.

**Results:**

A total of 2,195 patients were included, 1210 in the pre-intervention and 985 in the post-intervention cohort. After BPA implementation, 771/985 (78%) patients received a nasal MRSA PCR. Vancomycin was discontinued within 24 hours after initiation in 384/1210 (32%) patients pre-intervention and 559/985 (57%) patients post-intervention (adjusted OR 2.92 [95% CI 2.44 – 3.49], p < 0.001) (Table 1). Although “high risk” patient factors (e.g. need for mechanical ventilation) were associated with longer durations, vancomycin duration was significantly shorter in the post-implementation cohort for all “high risk” sub-groups (Figure 2).

**Figure:**
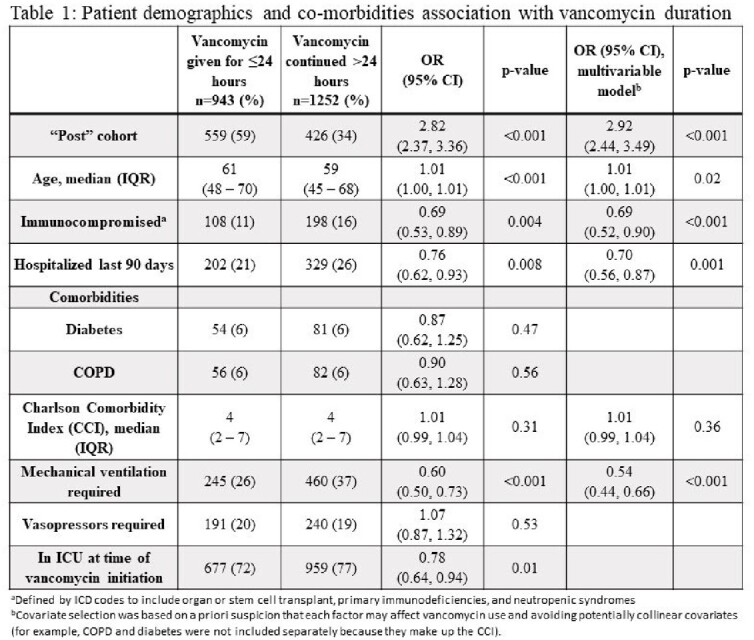

**Figure:**
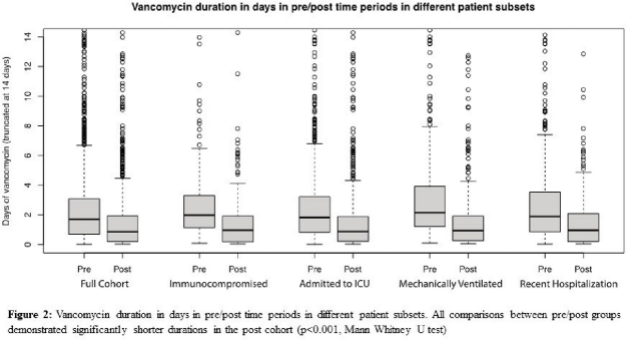

**Conclusion:**

BPA directed nasal MRSA PCR testing and vancomycin de-escalation effectively shortened duration of therapy among patients with suspected pneumonia. Utilizing a BPA directed approach can improve decision efficiency and allow valuable stewardship team resources to be allocated elsewhere.

**Disclosures:**

**All Authors**: No reported disclosures

